# Esophageal Replacement by Colonic Interposition for the Surgical Management of Acute Necrotic Gastric Volvulus: A Case Report

**DOI:** 10.7759/cureus.41257

**Published:** 2023-07-01

**Authors:** Steven Vuu, Samantha L Reiss, Lauren Aronson, Tovah Williamson, Darwin Ang

**Affiliations:** 1 General Surgery, University of Central Florida College of Medicine, Orlando, USA; 2 Medical School, University of Central Florida College of Medicine, Orlando, USA; 3 Surgery, University of Central Florida College of Medicine, Orlando, USA

**Keywords:** esophagectomy, gastric perforation, surgery, colonic interposition, gastric volvulus

## Abstract

Acute gastric volvulus, a condition where the stomach rotates around itself, is a rare clinical entity that requires prompt identification and immediate intervention to prevent life-threatening complications. Upon diagnosis, an emergent exploratory laparotomy is the procedure of choice, especially if complications, such as obstruction, ischemia, or perforation, are present. Management techniques and surgical corrections vary depending on the degree of obstruction, the consequent damage to surrounding structures, and the functional reservoir. We present a case of acute gastric volvulus with extensive esophageal and gastric necrosis requiring total gastrectomy and partial esophagectomy. We discuss the patient’s operative management requiring colonic interposition with esophagocolonic anastomosis to reconnect this patient’s gastrointestinal tract.

## Introduction

Gastric volvulus is a rare, potentially life-threatening condition in which the stomach abnormally rotates along one of its axes [1,2]. Primary gastric volvulus involves rotation due to intrinsic abnormalities of the gastric ligaments that result in laxity or lengthening, while the secondary form is more common and occurs from other anatomic abnormalities, such as paraesophageal hernias in adults or diaphragmatic hernias in children [2,3]. Gastric volvulus can be acute or chronic in nature with variable symptomatology, requiring a high index of suspicion upon initial presentation. Borchardt's triad - sudden onset epigastric pain, retching without vomiting, and inability to pass a nasogastric (NG) tube - is the classic presentation of complete volvulus, occurring in approximately 70% of acute symptomatic cases [1,2]. Complete outlet obstruction may lead to incarceration or strangulation, which can result in ischemia, necrosis, perforation, and shock. While upper gastrointestinal barium studies are considered the most sensitive and specific test for the diagnosis of gastric volvulus, they are seldom done. Abdominal computerized tomography (CT) is usually the preferred test of choice because of its speed, availability, and utility in pre-operative planning [1]. Upon evaluation, acute gastric volvulus may demonstrate a gastropyloric transition zone with an air-fluid level, abnormal antrum location, and signs of ischemia if present [4]. Gastric volvulus secondary to an inciting factor may also show structural abnormalities on imaging. 

Once the diagnosis of acute gastric volvulus is made, the patient is treated with emergent exploratory laparotomy especially if complications exist. After decompression and derotation of the stomach, nonsalvageable necrotic tissue is resected. When the extent of ischemia or perforation necessitates a total gastrectomy and partial esophagostomy, a jejunostomy tube is typically left in place, and reconstruction is deferred until the patient is stable [5]. Based on the functional reservoir, the patient may semi-electively undergo esophagojejunal anastomosis or colon interposition [5]. Many colonic interposition procedures are performed on pediatric patients. Although less common, esophageal substitution via colonic interposition is also a viable option for adult patients especially when the esophagus and jejunum are not sufficient in length to be bridged. In this report, we present a case of a 73-year-old female who presented with acute gastric volvulus and underwent esophageal replacement by colonic interposition.

## Case presentation

**Figure 1:**
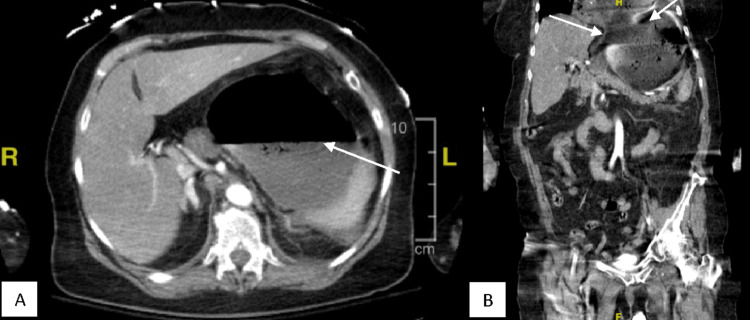
Preoperative CT scans: (A) gastric distension, air-fluid level, and adjacent inflammatory changes concerning for gastric volvulus; (B) paraesophageal hernia.

**Figure 2:**
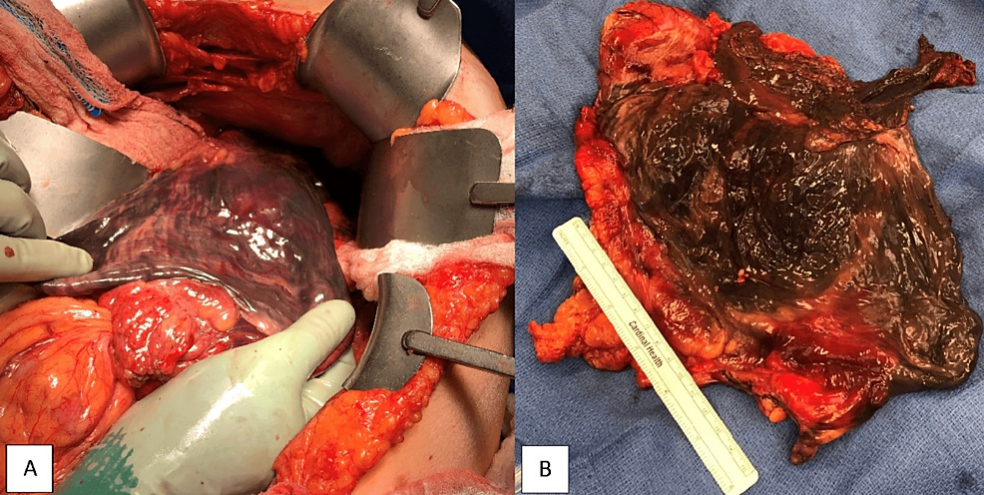
Necrotic stomach during the first exploratory laparotomy: (A) intraabdominal and (B) resected.

A 73-year-old female without a significant past medical history presented with severe epigastric pain, nausea, vomiting, and altered mental status for 24 hours. She was found to be in septic shock and was given antibiotics and a proton pump inhibitor intravenously. An anterolateral non-ST elevation myocardial infarction was suspected and later treated medically by cardiology, and the patient was intubated for respiratory failure. Her CT scans were concerning for acute gastric volvulus potentially due to a paraesophageal hernia (Figure 1). She was emergently taken to the operating room for an exploratory laparotomy. Intraoperatively, she was found to have a completely necrotic stomach, a necrotic distal esophagus from the gastroesophageal (GE) junction to 7 cm superior, and a 3 cm anterior perforation of the distal esophagus (Figure 2). A distal esophagectomy and total gastrectomy sparing the pylorus were performed with Echelon stapler green loads (Ethicon, Cincinnati, USA) to control the source of the necrosis, and a barker wound vacuum was placed.

Another exploratory laparotomy was done on day three of admission, after adequate resuscitation in the ICU, aiming to finalize the digestive system connection via a Roux-en-Y esophagojejunostomy procedure. The omental pedicle was mobilized, and the jejunum was divided 65 cm from the ligament of Treitz with a 60 blue load stapler (Ethicon, Cincinnati, USA). To aid in the mobilization of the duodenum and jejunum, the Cattell-Braasch and Kocher maneuvers were both utilized. An esophagojejunal anastomosis was attempted; however, the small bowel could not be brought up into the chest due to the limited mesenteric length. At this point, the decision was made to attempt a colonic interposition graft. The small bowel was returned to its normal anatomic position and re-anastomosed. To prepare a conduit for the next surgery, the transverse colon was divided between the right and left branches of the middle colic artery. Following division, the right colon, which was relying on the right branch of the middle colic artery, was dusky but had dopplerable pulses down to the terminal ileum. The abdomen was irrigated and closed with a temporary barker wound vacuum in order to re-assess the colon the next day to ensure its viability.

**Figure 3:**
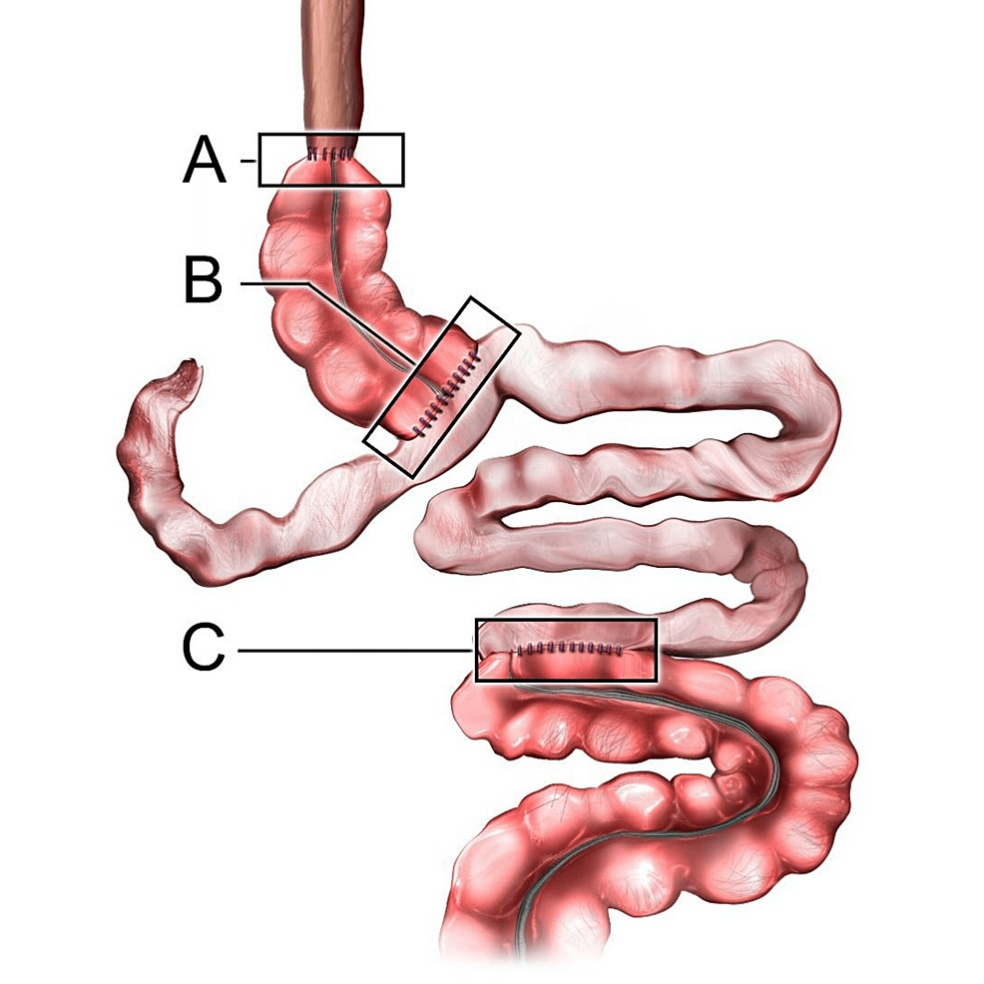
Illustration of the final sites of anastomoses: (A) esophagocolonic anastomosis, (B) colojejunal anastomosis, and (C) ileocolonic anastomosis. Image Credit: Ryan Dickerson (Educational Technology Department at University of Central Florida College of Medicine)

**Figure 4:**
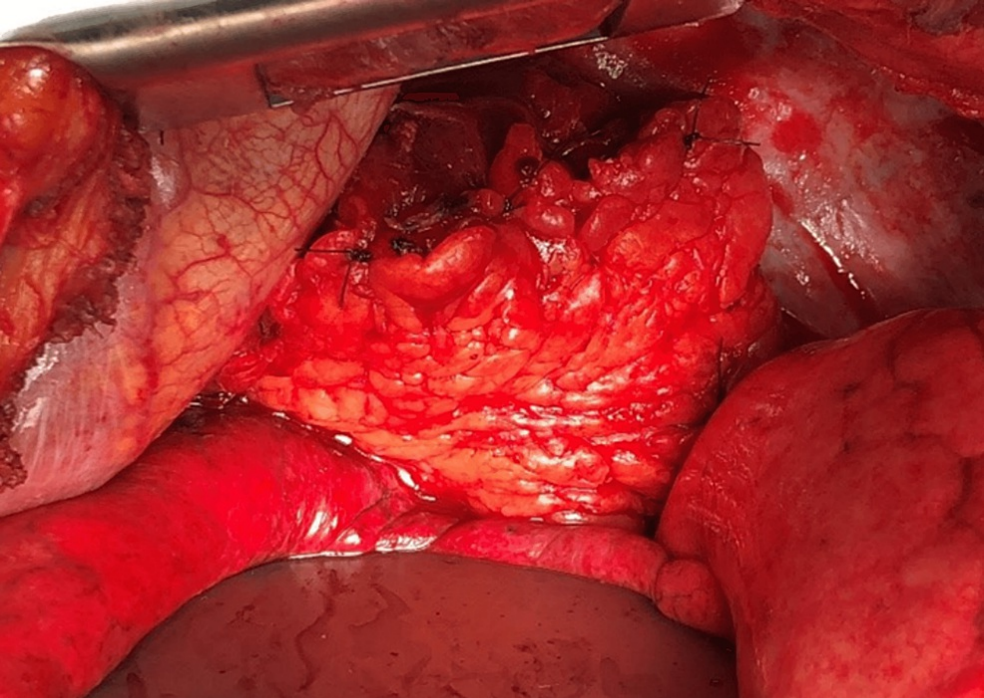
Esophagocolonic anastomosis with interrupted polydioxanone sutures covered with an omental patch.

Twelve hours later, on day four of admission, a final anastomosis was performed. Intraoperatively, the right colon was found to be engorged and edematous with good pulses on the Doppler ultrasound. The omental flap and distal esophagus were healthy, and the right lung was edematous. With the patient supine, a partial cecectomy and appendectomy was performed to decompress the colon. Afterward, the wood lamp test showed a good blood supply. The diaphragm hiatus was opened, and the cecum cephalad and omentum were mobilized toward the diaphragm hiatus into the chest. The abdomen was closed with a barker wound vacuum. The patient’s position was changed to left lateral decubitus for adequate exposure, and a right posterolateral thoracotomy was performed. The distal esophagus was identified and mobilized. The colon and omentum were brought up to the distal esophagus, and an esophagocolonic anastomosis was hand-sewn end to end with interrupted polydioxanone sutures (PDS). An nasogastric (NG) tube was placed past the anastomosis. Two 32 French chest tubes were placed, one over the anastomosis and one superiorly into the apex of the pleural space. Then, the barker wound vacuum was removed to provide access to the small and large intestines. A colojejunal anastomosis involving the loop of the jejunum and an ileocolonic anastomosis were both performed to reconnect the lower gastrointestinal tract. A feeding button loop jejunostomy tube was placed distally, and the abdomen was irrigated and closed. On postoperative day (POD) 1, the patient was started on trickle feeds. The total operative time for this three-phase surgery was approximately 18 hours. Figure 3 shows the final sites of anastomoses. Figure 4 shows the esophagocolonic anastomosis of the present case.

Unfortunately, the patient experienced a prolonged hospital stay and faced several complications. While intubated in the ICU, the patient also had worsening atelectasis on POD 6 that transformed into pneumonia with multidrug-resistant pseudomonas on bronchoalveolar lavage. The patient was treated with cefepime for seven days. For long-term respiratory support, a percutaneous tracheostomy on POD 10 was performed. Due to the need for long-term respiratory support, a percutaneous tracheostomy was performed on POD 10. In addition, the patient developed a left pleural effusion on two separate occasions, occurring five days and two months after the operation. The first episode was managed by placing a left pigtail catheter, while the second required a left chest tube.

Regarding the gastrointestinal aspect, an upper gastrointestinal series performed on POD 7 did not reveal any evident leaks. However, on POD 21, the patient returned to the operating room due to an increasing white blood cell count and the presence of greenish-brown contents leaking from the midline. An exploratory laparotomy was performed, and a leak or abscess in the right upper quadrant (RUQ) was identified and treated with washout and evacuation. Extensive abdominal adhesions were observed, with small bowel loops beginning to adhere together. In addition, an enteroatmospheric fistula was identified in the area of the bowel, which was repaired using silk stitches. However, due to the frozen abdomen, complete closure of the fascia without damaging the bowel was not possible. Drains were placed in the RUQ and left upper quadrant (LUQ). The fascia was partially closed, and a woven vicryl mesh was interposed between the fasciae over the small bowel. A white and black sponge wound vacuum-assisted closure (VAC) were applied on the top of the mesh.

On POD 33, a repeat abdominal wound washout was performed, during which a Malecot drain was inserted into the fistula to manage the output. A woven vicryl mesh was placed over the surrounding area of the small bowel, and the white and black sponges were positioned on the mesh around the Malecot drain. On POD 56, a CT-guided drain was placed to address concerns of an RUQ abscess. A J-tube study on POD 78 indicated no leaks and confirmed good positioning, while a CT scan did not reveal any acute findings. As of POD 87, the patient remained in long-term acute care, receiving total parenteral nutrition and prophylactic nasogastric tube suction. She had a right chest tube and an abdominal wound vacuum in place.

## Discussion

In the presented case, colonic interposition was performed, involving esophagocolonic, colojejunal, and ileocolic anastomoses, within 72 hours after the discovery of a necrotic esophagus and stomach. Colonic interposition is a suitable option for esophageal replacement when anatomical limitations prevent less invasive gastrointestinal tract reattachment [6]. In this patient, initial attempts were made to perform a Roux-en-Y esophagojejunostomy after resecting the distal esophagus and stomach. However, this procedure was not feasible due to the inability to reach the distal esophagus and jejunum. Therefore, colonic interposition was the optimal surgical approach as it provided the necessary length for gastrointestinal tract reconnection [7].

Colonic interposition is a relatively uncommon procedure. Although rare, it is often indicated for pediatric patients with esophageal atresia and adult patients with esophageal malignancy. It is also used in both pediatric and adult cases to treat benign esophageal strictures resulting from caustic substance ingestion or gastroesophageal reflux disease [6,8]. When fistulas and esophageal or surrounding tissue necrosis are present, more invasive gastrointestinal reattachment methods, such as colonic interposition, may be required [6].

Typically, colonic interposition involves anastomosis of the colon conduit to the remaining part of the stomach [7]. However, in this case, total gastrectomy was performed, making gastric utilization for gastrointestinal tract reconstruction impossible. Therefore, the ascending colon conduit was interposed between the proximal esophagus and jejunum. Colonic interposition can involve either the ascending or descending colon [7,9]. In this case, the anastomosis was created between the esophagus and the right colon, specifically the cecum. This choice was made to maintain consistent peristalsis in the same direction, from the esophagus to the colon. Performing the anastomosis with the descending colon would not allow for this continuity of peristalsis [7,9].

Postoperative complications of colonic interposition include, but are not limited to, stricture formation, anastomotic leakage, and fistula formation [6,8,10]. In this patient, interrupted PDS sutures were used instead of the Lembert stitch to reduce the risk of strictures. The rate of these complications when using the Lembert stitch has not been extensively studied; however, the literature suggests a higher rate of anastomotic leakage and strictures with the Lembert stitch compared to interrupted sutures [10]. Although the patient did not develop strictures, there was suspicion of an anastomotic leak, and an enteroatmospheric fistula (EAF) was identified on POD 21. The requirement for multiple exploratory laparotomies and gastrointestinal resections in this patient increased the risk of EAF formation and other postoperative complications [11].

We discussed this case at our Morbidity and Mortality Conference in our residency program. In retrospect, considering the patient's prolonged hospital stay and complications, an alternative treatment plan could have involved resecting the necrotic esophagus and stomach, followed by the placement of a feeding jejunostomy tube and NG tube for suction. This approach would have allowed the patient to remain in discontinuity for several weeks, giving her time to recover before undergoing the colonic interposition. However, it is important to note that, at that time, the patient was not on vasopressors and had been adequately resuscitated. Our rationale was to facilitate a faster recovery and earlier resumption of oral intake. Nevertheless, for future similar cases involving elderly patients, it might be more prudent to delay the anastomosis to allow for a longer recovery period.

## Conclusions

Colonic interposition with esophagocolonic anastomosis is a feasible option for acute gastric volvulus complicated by extensive unviable tissue. This patient had multiple surgeries, complications, and prolonged hospital stay. These are not minimal postoperative sequelae. However, in patients with extensive necrosis due to gastric volvulus, colonic interposition remains a viable treatment option.

## References

[1] Rashid F, Thangarajah T, Mulvey D, Larvin M, Iftikhar SY (2010). A review article on gastric volvulus: a challenge to diagnosis and management. Int J Surg.

[2] Verde F, Hawasli H, Johnson PT, Fishman EK (2019). Gastric volvulus: unraveling the diagnosis with MPRs. Emerg Radiol.

[3] Shivanand G, Seema S, Srivastava DN, Pande GK, Sahni P, Prasad R, Ramachandra N (2003). Gastric volvulus: acute and chronic presentation. Clin Imaging.

[4] Millet I, Orliac C, Alili C, Guillon F, Taourel P (2014). Computed tomography findings of acute gastric volvulus. Eur Radiol.

[5] Pandey S, Paudel M, Parajuli A, Ghimire R, Neupane A (2021). Mesenteroaxial gastric volvulus: a case report. JNMA J Nepal Med Assoc.

[6] Thomas P, Fuentes P, Giudicelli R, Reboud E (1997). Colon interposition for esophageal replacement: current indications and long-term function. Ann Thorac Surg.

[7] Bakshi A, Sugarbaker DJ, Burt BM (2017). Alternative conduits for esophageal replacement. Ann Cardiothorac Surg.

[8] Coopman S, Michaud L, Halna-Tamine M, Bonnevalle M, Bourgois B, Turck D, Gottrand F (2008). Long-term outcome of colon interposition after esophagectomy in children. J Pediatr Gastroenterol Nutr.

[9] Fürst H, Hartl WH, Löhe F, Schildberg FW (2000). Colon interposition for esophageal replacement: an alternative technique based on the use of the right colon. Ann Surg.

[10] Feng F, Sun L, Xu G (2015). Albert-Lembert versus hybrid-layered suture in hand sewn end-to-end cervical esophagogastric anastomosis after esophageal squamous cell carcinoma resection. J Thorac Dis.

[11] Haack CI, Galloway JR, Srinivasan J (2014). Enterocutaneous fistulas: a look at causes and management. Curr Surg.

